# Immunoaffinity extraction followed by enzymatic digestion for the isolation and identification of proteins employing automated μSPE reactors and mass spectrometry

**DOI:** 10.1007/s00216-022-04381-0

**Published:** 2022-11-12

**Authors:** Karen Duong, Simin Maleknia, David Clases, Andrew Minett, Matthew P. Padula, Philip A. Doble, Raquel Gonzalez de Vega

**Affiliations:** 1 The Atomic Medicine Initiative, University of Technology Sydney, Ultimo, Australia; 2 School of Mathematical and Physical Sciences, Faculty of Science, University of Technology Sydney, Ultimo, Australia; 3 Nano Micro LAB, Institute of Chemistry, University of Graz, Graz, Austria; 4 ePrep Pty Ltd, Oakleigh, Victoria 3166 Australia; 5 School of Life Sciences and Proteomics Core Facility, Faculty of Science, University of Technology Sydney, Ultimo, Australia; 6 TESLA-Analytical Chemistry, Institute of Chemistry, University of Graz, Graz, Austria

**Keywords:** IMER, Immobilised enzyme, Immunosorbent, Protein analysis, μSPE, Mass spectrometry

## Abstract

This work describes a novel automated and rapid method for bottom-up proteomics combining protein isolation with a micro-immobilised enzyme reactor (IMER). Crosslinking chemistry based on 1-ethyl-3-(3-dimethylaminopropyl) carbodiimide/*N*-hydroxysuccinimide (EDC/NHS) coupling was exploited to immobilise trypsin and antibodies onto customisable silica particles coated with carboxymethylated dextran (CMD). This novel silica–CMD solid-phase extraction material was characterised using Fourier transform infrared spectroscopy (FTIR), scanning electron microscopy (SEM), conductometric titrations and enzymatic colorimetric assays. Micro-solid-phase extraction (μSPE) cartridges equipped with the modified CMD material were employed and integrated into an automated and repeatable workflow using a sample preparation workstation to achieve rapid and repeatable protein isolation and pre-concentration, followed by tryptic digestion producing peptide fragments that were identified by liquid chromatography mass spectrometry (LC-MS).

**Graphical abstract:**

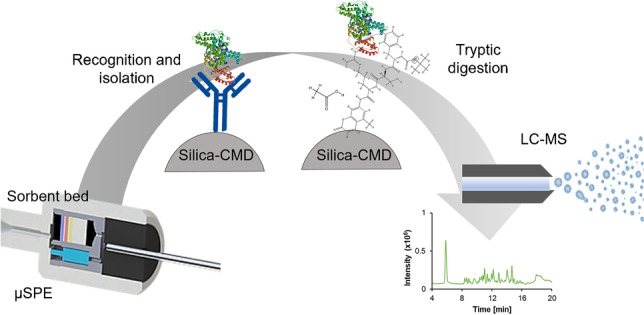

**Supplementary Information:**

The online version contains supplementary material available at 10.1007/s00216-022-04381-0.

## Introduction

Targeted proteomics and low-level quantification of proteins requires purification and pre-concentration followed by proteolytic digestion to generate peptides that may be easily measured and identified by mass spectrometry. The control of selectivity during sample pre-treatment is often performed using a sorbent containing immobilised antibodies that present a high affinity towards a target analyte. Following selective immunoextraction, an enzymatic digestion is then undertaken in solution. Conventional proteolytic digestions of isolated proteins may be subject to long digestion times, unwanted enzyme autolysis, low activity, poor reproducibility and difficultly of automation [1, 2]. To overcome these drawbacks, the proteolytic enzymes may be immobilised on a solid support and integrated into an analytical workflow, allowing total automation of the analysis [3, 4]. Affinity and enzymatic reactors have been widely used to improve selectivity during sample pre-treatment whilst reducing digestion times, demonstrating promising advances for automation and higher sample throughput. However, only a few studies have presented the combination of both approaches. Hoos et al. reported an online combination of immunoaffinity chromatography for selective sample pre-treatment followed by solution phase digestion for the detection of covalent adducts of human serum albumin (HSA) [5]. Freije et al. [6] developed an automated and integrated system comprising an immobilised inhibitor cartridge for activity-dependent enrichment followed by an immobilised trypsin reactor for detection of MMP12 spiked into urine samples. Bonichon et al. reported the use of an immunosorbent coupled online to a pepsin IMER (immobilised enzyme reactor) for the total online analysis of HuBuChE-OP adducts from plasma [7]. The same group exploited the online method to investigate the potential of three monoclonal antibodies (mAb2, 3E8 and B2 18-5), grafted on sepharose, to extract HuBuChE from plasma [8]. More recent studies have also shown the development of automated platforms which integrate the multiple steps of the protein sample preparation workflow including protein depletion, fractionation, denaturation, digestion and peptide enrichment into an integrated sample preparation system allowing increased throughput [9, 10].

The choice of an adequate support material is critical for selective immobilisation of a biomolecule. Specifically, the material must be suitable for covalent functionalisation, provide thermal and mechanical resistance and be biocompatible. Ideally, the material should be able to be regenerated for reusability, be stable under reaction conditions and be relatively inexpensive and environmentally friendly [11]. Organic–inorganic hybrids have excellent potential as support materials for enzymes providing enhanced stability, mechanical resistance and compatibility with biological molecules. These support materials comprise inorganic materials, such as silica, inorganic oxides, minerals, carbon materials and magnetic nanoparticles coated with polymers or polysaccharides, such as chitosan [12], poly(ethylene glycol) (PEG) [13], cellulose [14] or dextran [15]. Polysaccharides are effective for the reduction of protein and silica surface interactions and for suppressing unwanted non-specific binding due to increased hydrophilicity of the carrier [16]. It is also possible to chemically modify the support surface to enable numerous immobilisation techniques. Several methods for enzyme immobilisation have been proposed [17], including enzyme entrapment, crosslinking, adsorption, covalent immobilisation and affinity. Among all these methods, covalent immobilisation generally ensures the highest strength of the bonding between support and enzyme, minimising leakage and wash-off issues. Similarly, the immobilisation of antibodies on the bioanalytical platforms is the most critical step as it directly affects analytical performance [18]. This immobilisation may be performed via a wide range of strategies such as physical adsorption, orientated binding by intermediate proteins, covalent binding, biotin–avidin interactions, affinity tags and site-specific binding [19, 20]. It has been recognised that covalent immobilisation is preferred when the coated substrate is subjected to flow or extended times in solution as leaching of the immobilised molecule from the substrate is prevented [21]. The most widely used covalent binding strategy is the heterobifunctional crosslinking of the amino or carboxyl groups on antibodies to the free carboxyl or amino groups on bioanalytical platforms using 1-ethyl-3-(3-dimethylaminopropyl) carbodiimide (EDC) along with N-hydroxysuccinimide (NHS) or sulfo-NHS [22, 23]. The high specificity of antibodies to target analytes renders immunoextraction useful for isolation and enrichment of analytes occurring at very low concentration levels in environmental and biological samples with very complex matrices.

This work details the fabrication of a new inorganic–organic solid-phase support material for the in situ immobilisation of biomolecules. To assess the desired customised support material, several experiments were required including validation of the material and its suitability for trypsin conjugation and antibody immobilisation. Aminopropyl silica (APS) with a particle size of 3 μm was used as an inorganic solid-phase support material. To increase biocompatibility, hydrophilicity and functionality of the surface, silica particles were modified with carboxymethylated dextran (CMD). This customisable material was packed into micro-solid-phase extraction (μSPE) cartridges for accurate and reproducible automated methods of protein isolation and pre-concentration, followed by trypsin digestion to produce peptide fragments that were identified by liquid chromatography mass spectrometry (LC-MS).

## Experimental

### Chemicals and consumables

Aminopropyl silica (APS) (3 μm, 120 Å) was obtained from Osaka Soda (Amagasaki, Hyogo, Japan). Carboxymethyl-dextran sodium salt (CMD), 1-ethyl-3-(3-dimethylaminopropyl) carbodiimide (EDC), *N*-hydroxysuccinimide (NHS), ethanolamine, sodium hydroxide pellets, potassium hydrogen phthalate (KHP), 2-(*N*-morpholino)ethanesulfonic acid (MES), phosphate buffered saline (PBS) pH 7.4 solution, 2,2′-azino-bis(3-ethylbenzothiazoline-6-sulfonic acid) liquid substrate (ABTS), horseradish peroxidase (HRP), trypsin from bovine pancreas (TPCK treated), bovine serum albumin (BSA), equine cytochrome c (Cyt c), human serum (H4522), Tris base, urea, iodoacetamide (IAA), dithiothreitol (DTT), Nα-benzoyl-l-arginine ethyl ester (BAEE) and formic acid 98% were purchased from Sigma Aldrich (Castle Hill, NSW, Australia). Anti-BSA monoclonal antibody (ab9092) was purchased from Abcam (Melbourne, Victoria, Australia). Mouse IgG VisUCyte HRP polymer antibody was obtained from R&D Systems (Minneapolis, MN, USA). Acetonitrile 99.9% purity (ACN) was obtained from Chem-Supply (Gillman, South Australia, Australia). Ultra-pure water (18.2 MΩ cm) obtained from a Sartorius 611 Arium® pro water generation system was used for all preparation and dilutions unless stated otherwise.

### Sample preparation

#### Protein reduction and alkylation

BSA protein solutions were prepared by dissolving 10 mg of protein in 1 mL of 100 mM Tris buffer containing 6 M Urea. 5 μL of DTT (200 mM in 100 mM Tris) were added to 100 μL of the protein solution, and samples were reduced at room temperature for 1 h and subsequently alkylated with 20 μL of IAA (200 mM in 100 mM Tris) for 1 h. Additionally, 20 μL of DTT were added to consume any unreacted IAA. Finally, 855 μL of ultra-pure water were added to the mixture to decrease the urea concentration below 1 M. Cyt c protein samples were digested without any further pre-treatment.

#### In-solution digestion

To a 50 μL of protein solution, 47 μL of 500 mM Tris buffer pH 8 were added, followed by 2 μL of 5 ng/μL trypsin (1:200 enzyme to protein ratio). Samples were incubated at 37 °C for 18 h, and 1 μL of concentrated formic acid was added to stop digestion.

#### Immunoaffinity extraction followed by enzymatic digestion

20 ng/μL of BSA were spiked onto a human serum sample. 100 μL of this solution were then added to a 1.5-mL microcentrifuge tube, and human serum sample was reduced and alkylated followed the same protocol as BSA.

### Micro-solid-phase extraction (μSPE)

The μSPE procedure was developed with a digiVOL® Programmable Digital Syringe Driver using ePrep® micro-solid-phase extraction cartridges (μSPEed) obtained from ePrep (Oakleigh, Victoria, Australia) and packed with the novel generic customisable material (see Fig. S1). μSPEed cartridges have two flow paths: the first flow path is designed for the direct aspiration of liquids into the syringe barrel using a one-way check valve, and the second flow path dispenses the liquid through the sorbent bed with a low-dead volume connection. Compared to traditional SPE where 40-50-μm particles are typically used, μSPEed cartridges use small sorbent particle sizes of 3 μm to increase the surface and operation at higher pressures [24], allowing more efficient digestions, faster separation of target analytes and enhanced reproducibility. Once the μSPEed workflows were developed and optimised, they were moved over to the ePrep Sample Preparation Workstation for automation (see Fig. S1).

### Development of customisable material

The customisable material was manufactured from aminopropyl silica beads modified with a CMD coating exploiting carbodiimide crosslinking chemistry. In a reaction vessel, 100 mg of CMD, 40 pmol of EDC, 80 pmol of NHS and 10 mL of binding buffer (50 mM MES) were mixed for 2 h at room temperature. The solution was then mixed with 100 mg of APS and allowed to react overnight at room temperature (1:1 ratio of CMD to APS by weight). The resulting material (silica–CMD) was vacuum-filtered, washed with MQ water and allowed to dry. A Nicolet 6700 Fourier transform infrared spectrometer (FTIR) (Thermo Fisher, Waltham, MA, USA) using attenuated total reflectance (ATR) was used to characterise the CMD modification. Scanning electron microscopy (SEM) images were taken at 3 kEV using the Zeiss Supra 55 VP (Carl Zeiss AG, Oberkochen, Germany). Conductometric titrations were performed using a Hach HQ14D meter (Hach, Dandenong South, Victoria, Australia) to determine the concentration of carboxylate groups on the novel material. Based on a method previously described by Wu et al. [25], 100 mg of material were suspended into 100 mL of deionised water using ultrasonication; 200 μL of 0.10 M HCl were mixed into the suspension and was titrated under nitrogen against 25 mM of NaOH. To determine the exact NaOH concentration, it was titrated against three aliquots of KHP prepared in ultra-pure water.

To confirm the covalent binding of biomolecules to the novel material, silica–CMD was packed into μSPEed cartridges, and HRP was immobilised as a model enzyme, which catalyses the colorimetric reaction of ABTS in the presence of hydrogen peroxide. The formation of an ABTS cation radical was spectrophotometrically monitored using a Thermo Fisher Multiskan Ascent 96/384 Plate Reader (Thermo Fisher Scientific, North Ryde, NSW, Australia) [26]. For covalent binding of HRP, μSPEed cartridges were conditioned with 500 μL of 10 mM MES buffer, activated with 0.1 M EDC/NHS in 10 mM MES, loaded with 50 μg of HRP and end-capped with 0.1 M ethanolamine pH 8. The cartridge was then washed with 500 μL of PBS (1×) buffer prior to loading the 100 μL of ABTS liquid substrate. The loading and colorimetric reaction of ABTS was repeated 10 times within the same cartridge, and fractions were collected in a microwell plate and scanned.

### Immobilised enzyme reactor (IMER) through μSPEed cartridges

For the construction of a trypsin IMER, μSPEed cartridges were packed with silica–CMD material, and trypsin was immobilised in-situ. An immobilisation buffer was prepared using MES at a final concentration of 10 mM. A 500 mM tris buffer solution (pH 8) was used to prepare three buffers, a running buffer used as a diluent for protein samples and cartridge conditioning (25 mM Tris with 0.5 mM CaCl_2_), a salt wash buffer (25 mM Tris with 10 mM CaCl_2_) and an elution buffer (25 mM Tris with 10 mM CaCl_2_ and 10% ACN). For end-capping, ethanolamine (0.1 M) was prepared and adjusted to pH 8 with concentrated hydrochloric acid. BSA and Cyt c were chosen as model proteins. BSA was denatured, reduced and alkylated with urea, DTT and IAA, whilst Cyt c was prepared in 500 mM Tris buffer pH 8. Prior to digestion, all protein samples were diluted to working concentrations with the running buffer.

In-situ immobilised trypsin was prepared directly in the μSPEed cartridges using the digiVOL® digital syringe driver. The cartridge was conditioned twice with 500 μL of 10 mM MES buffer and activated with 0.1 M EDC/NHS in 10 mM MES. The cartridge was then loaded with 500 μL of 50 ng/μL trypsin in 10 mM MES buffer and end-capped with 0.1 M ethanolamine at pH 8. Between each step, cartridges were washed with 500 μL of 10 mM MES buffer. Finally, to remove any non-covalently bound trypsin, the cartridge was washed three times with 500 μL salt wash buffer (25 mM Tris with 10 mM CaCl_2_).

For protein digestion, the prepared cartridges were conditioned twice with 500 μL of running buffer before loading the protein samples. 100 μL of protein sample (10 ng/μL) were loaded onto the cartridge and cycled through various times for different digest contact times ranging from 5 to 10 min. To recover generated protein fragments, 100 μL of elution buffer were passed through the cartridge, collected and spiked with 1 μL of concentrated formic acid for LC-MS analysis.

To investigate the cartridge’s digest performance, in-situ immobilised trypsin cartridges were prepared to perform digests of 1000 μM BAEE prepared in PBS (1×) buffer. Each cartridge was conditioned with 2 × 500 μL salt buffer and 2 × 500 μL PBS (1×) buffer. The BAEE solution was then cycled through allowing 5 min of contact time with the trypsin cartridge before elution into an LC vial (15 cycles of 100 μL at 300 μL/min). A further 100 μL of 1% formic acid were passed through the cartridge into the LC vial to dilute the sample 1 in 2 and quench the enzymatic cleavage.

### Covalent immobilisation and antibody-antigen (Ab-Ag) complex formation

**Fig. 1:**
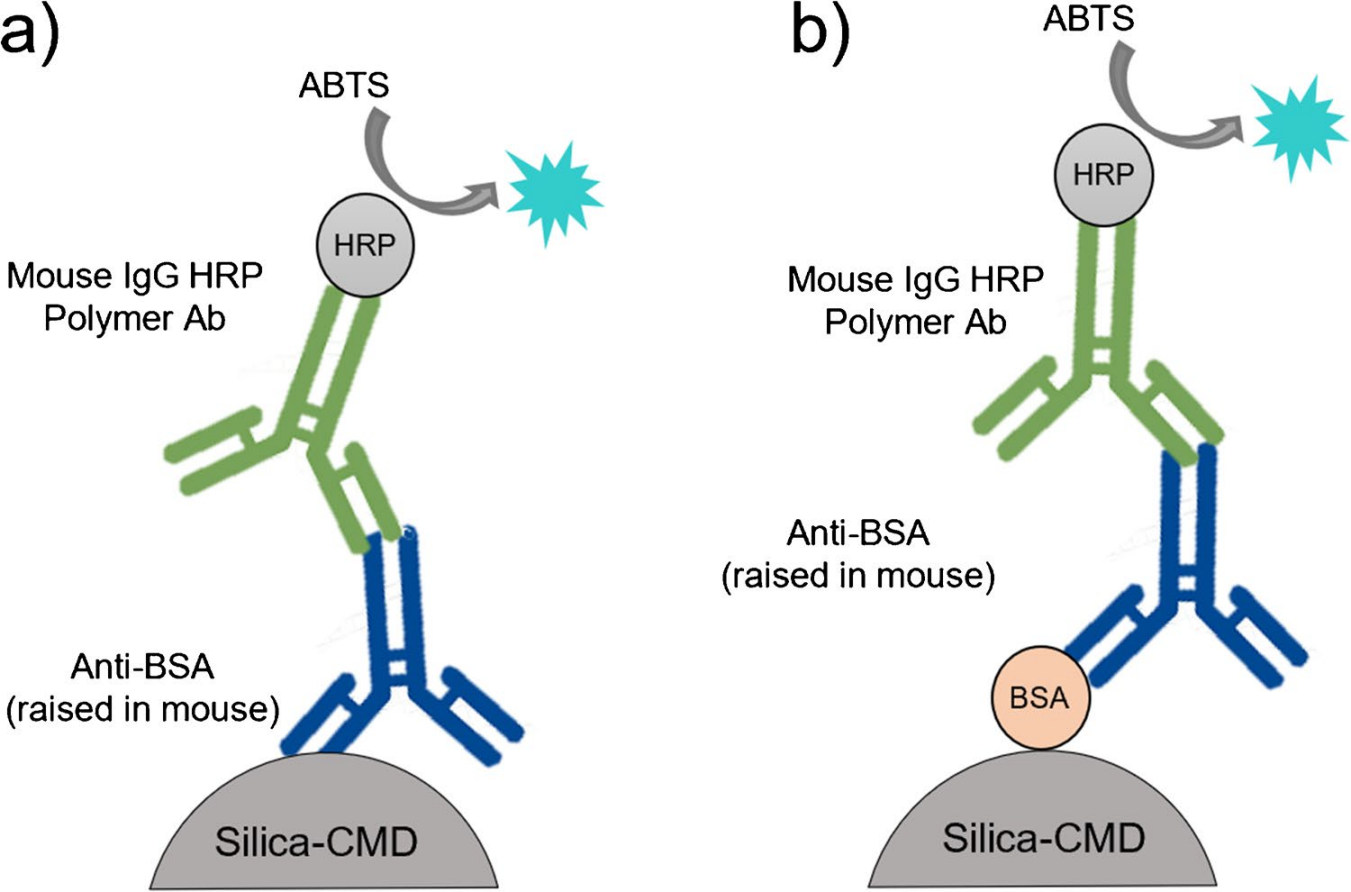
Covalent immobilisation of anti-BSA (**a**) and BSA (**b**) onto the silica–CMD particles. A secondary antibody labelled with HRP was used to confirm the binding of both anti-BSA and BSA via a colorimetric reaction

To ensure covalent immobilisation of the antibody onto silica–CMD, a colorimetric visualisation using HRP was performed. Approximately, 10 mg of loose silica–CMD were added to an Eppendorf tube followed by 200 μL of 10 mM MES buffer, 10 μg of anti-BSA Ab, 1 mg of NHS and 2 mg of EDC and allowed to react for 1 h. The material was washed with 500 μL of PBS buffer by a vortex, spun down with a microcentrifuge and the solution decanted off. This was performed 5 times with PBS (10×) followed by 5 times with PBS (1×). 20 μL of mouse IgG HRP polymer were then incubated with the material for 30 min to bind anti-BSA Ab (raised in mouse). The material was then washed with 10 cycles of PBS (1×) to remove excess mouse IgG HRP polymer before adding 200 μL of ABTS liquid substrate for visualisation (see Fig. 1a). In addition, a second colorimetric visualisation using HRP was used to ensure Ab–Ag complex binding. BSA was covalently bound to silica–CMD material, incubated with anti-BSA and mouse IgG HRP polymer and visualised using the ABTS substrate (see Fig. 1b).

### Antibody immobilisation onto μSPEed cartridges for protein isolation

Antibodies were covalently immobilised onto silica–CMD μSPEed cartridges in-situ using the digiVOL® digital syringe driver. An immobilisation buffer was prepared using MES to a final concentration of 10 mM. Tris buffer was prepared from tris base at a concentration of 500 mM and adjusted to pH 8 with HCl. Tris buffer was used to prepare two buffers, a running buffer to be used as a diluent for protein samples and cartridge conditioning (25 mM Tris with 1 mM NaCl) and a salt wash buffer (25 mM Tris with 20 mM NaCl). The cartridge was conditioned twice with 500 μL of 10 mM MES buffer activated with 0.1 M EDC/NHS in 10 mM MES, loaded with 20 ng/μL of antibody in 10 mM MES and end-capped with 0.1 M ethanolamine pH 8 with a 500 μL wash with 10 mM MES between each step. The cartridge was then washed three times with 500 μL wash buffer to remove any non-specifically bound antibody and conditioned twice with 500 μL running buffer. The sample prepared in running buffer was loaded and cycled twice, washed three times with 500 μL wash buffer to remove all non-specifically bound components and eluted with 80 μL of 0.2% HCl into an LC vial. 20 μL of 500 mM Tris 2.5 mM CaCl_2_ pH 8 were add to the elute for compatibility with trypsin digestion. The cartridge was immediately reconditioned to pH 8 twice with 500 μL of wash buffer to prevent antibody denaturation for further use.

### Instrumentation

The immobilisation of trypsin using the BAEE substrate was confirmed conducting *HPLC-UV* separations (Thermo Fisher Ultimate 3000, North Ryde, NSW, Australia). A Zorbax 300SB-C8 column (2.1 × 100mm, 1.8 μm) (Agilent Technologies, Santa Clara, CA, USA) was used for separation. The mobile phase was 85% water and 15% acetonitrile containing 0.1% formic acid. The flow rate was 0.2 mL/min, and the injection volume 1 μL. UV detection was set at 254 nm.

*Liquid chromatography quadruple time-of-flight mass spectrometry (LC-QToF-MS)* experiments for protein analyses were performed using a 1290 Infinity II LC equipped with an Accucore C18+ column (2.1 × 100 mm, 1.5 μm) and coupled to a 6510 series QToF-MS (Agilent Technologies, Santa Clara, CA, USA). The mobile phases were water (A) and acetonitrile (B) both containing 0.1% formic acid. The gradient program (A:B v/v) was 95:5 from 0 to 20 min followed by a linear gradient until 40:60 at 25 min, which was held for 5 min. The flow rate was 0.2 mL/min, and the injection volume was 20 μL. MS parameters were optimised in positive electrospray ionisation (ESI). The optimised values for capillary and fragmentor voltages were 3500 V and 175 V, respectively, and the drying gas flow was set to 5 L/min at 325 °C. The mass range analysed was 400–2000 m/z.

MS data obtained by LC-QToF-MS was processed using Agilent MassHunter B.07.00 (Agilent Technologies) to obtain a peptide list of charge state ≥2+. The mass list was searched against the contaminants database using MASCOT Peptide Mass Fingerprint (Matrix Science). Within the MASCOT search engine, the enzyme selected was trypsin, and experimental ion peptide tolerance was set to ±1.2 Da. The criteria also allowed two missed cleavages to account for incomplete digestion.

*Liquid chromatography LTQ-Orbitrap mass spectrometry (LC-LTQ-Orbitrap MS)* experiments for protein analyses were carried out using a Waters ACQUITY M-Class Nano LC (Waters, Rydalmere, NSW, Australia) coupled to Thermo Fisher Q Exactive Plus (Thermo Fisher Scientific, North Ryde, NSW, Australia). Chromatographic separation was performed using a nanoEase Symmetry C18 trapping column in line with a PicoFrit column (300 mm × 75 μm (ID); New Objective, Woburn, MA) packed with Magic C18AQ resin (3 μm, Michrom Bioresources, Auburn, CA). Elution solvents were 2% water (A) and 98% acetonitrile containing 0.2% formic acid (B). 5 μL of sample were loaded at 15 μL/min for 3 min onto the trap column and then washed into the PicoFrit column using the following program, 0–15 min, 5 to 30%B, and 15–28 min, 30–80%B, held for 5 min at 80%B followed by a re-equilibration step for 2 min with 5%B. The eluting peptides were ionised at 2400 V. A data-dependant MS/MS (dd MS2) experiment was performed, with a survey scan of 350–1500 Da performed at a mass resolution of 70,000 for peptides of charge state 2+ or higher with an automatic gain control (AGC) target of 3e^6^ and maximum injection time of 50 ms. The top 12 peptides were selected and fragmented in the higher-energy collisional dissociation (HCD) cell using an isolation window of 1.4 m/z, an AGC target of 1e^5^ and maximum injection time of 100 ms. Fragments were detected with a mass resolution of 17,500, and the product ion fragment masses were measured over a mass range of 120–2000 Da.

MS/MS data files obtained by LC-LTQ-Orbitrap MS were searched using Peaks Studio X against either the human or bovine proteome and a list of common contaminants with the following parameter settings. Within the Peak Studio X search engine, the enzyme selected was trypsin, variable modifications were specified as carbamidomethyl (C), deamidation (N and Q) and oxidation (M), and the precursor and fragment ion peptide mass tolerance was set to 10ppm and ±0.05 Da, respectively. The criteria also allowed three missed cleavages to account for incomplete digestion. The results of the search were then filtered to include peptides with a −log10P score that was determined by the false discovery rate (FDR) of <1%).

## Results and discussion

### Characterisation of the support material

**Fig. 2:**
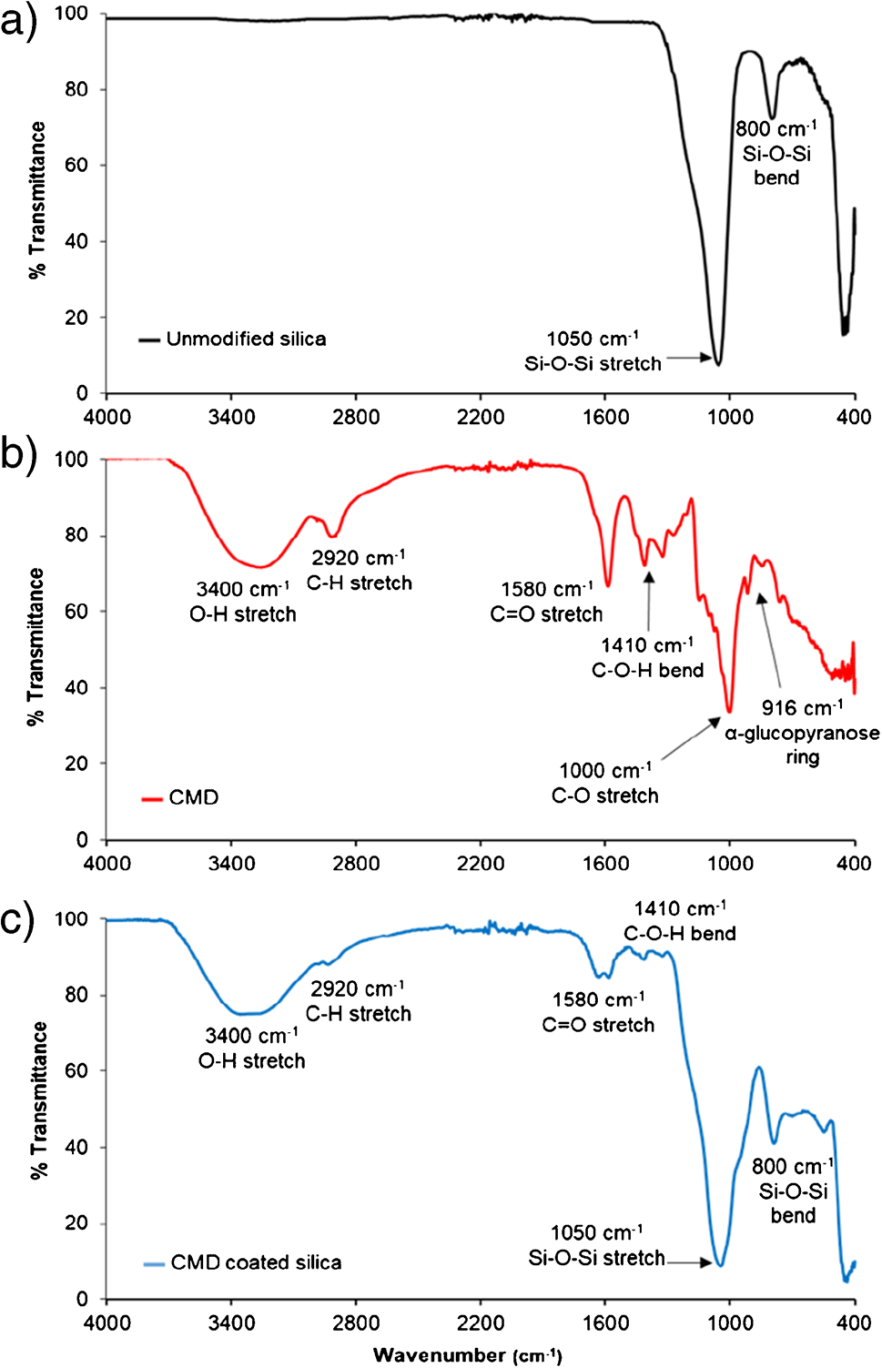
FTIR absorption spectra for unmodified silica (**a**), CMD (**b**) and the CMD coated silica (**c**). A combination of absorption band visible in unmodified silica and CMD confirms the formation of the desired silica–CMD material

Organic–inorganic hybrid support materials for IMERs have excellent stability and compatibility with appropriate functional groups for facile biomolecule immobilisation. We fabricated 3-μm carboxymethylated dextran (CMD) covered silica support particles for covalent binding of biomolecules exploiting an EDC/NHS coupling chemistry. This polysaccharide-coated surface is also known to suppress non-specific binding [16]. The silica–CMD material was characterised by FTIR, SEM and conductometric titration to verify the integrity of the CMD coating and to estimate binding capacity. FTIR was employed to ensure successful coating of CMD by interrogation of the absorption bands of functional groups associated with both the silica support material and the CMD. Figure 2a shows the FTIR absorption spectra of unmodified silica with two absorption bands at wavenumbers of 1050 cm^−1^ and 800 cm^−1^ corresponding to the Si-O-Si stretch and trend vibrations [27]. Figure 2b shows the absorption bands for CMD (O-H stretch 3400 cm^−1^, C-H stretch 2920 cm^−1^, C=O stretch 1580 cm^−1^, C-O-H bend 1410 cm^−1^, C–O stretch 1000 cm^−1^, α-glucopyranose ring 916 cm^−1^ [28]). The successful formation of the CMD coated silica support material was apparent in Fig. 2c, where a combination of all absorption bands was observed.

**Fig. 3:**
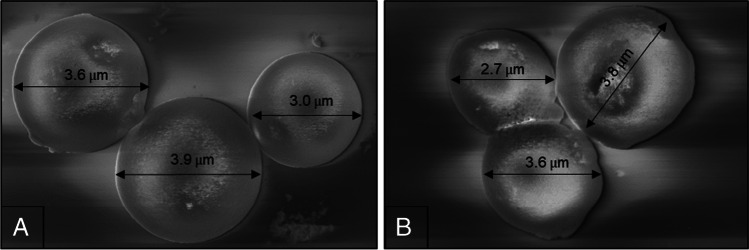
SEM images of the silica bead particles before (**A**) and after (**B**) modification with the CMD coating

SEM was employed to confirm the integrity and shape following coating. This was critical as particle shape influences surface area, packing density and therefore efficiency. Figure 3 shows magnified images of the silica particles’ size and shape, showing no substantial differences in their physical appearance before and after modification with CMD.

**Fig. 4:**
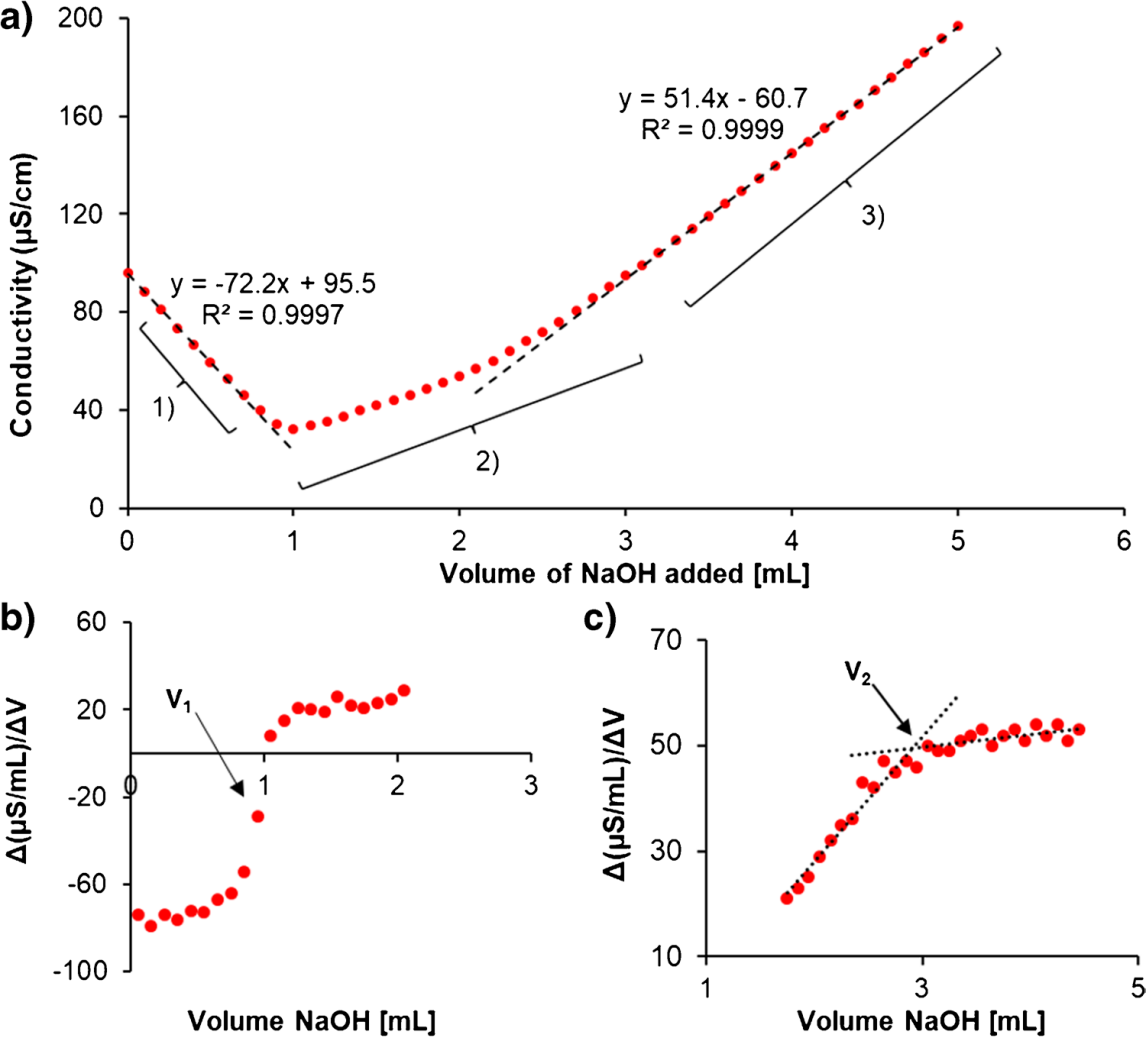
**a** Conductometric titration of silica–CMD material showing three stages of the titration: (1) neutralisation of HCl by NaOH, (2) neutralisation of acidic -COOH groups on the silica–CMD material and (3) excess additions of NaOH following neutralisation of all acidic groups. The first derivatives of the titration show the **b** starting and **c** end point of the neutralisation of -COOH groups on the silica–CMD which is observed in the second stage of the titration

A conductometric titration was performed to quantify the number of -COOH groups available for enzyme conjugation, as shown in Fig. 4a. The titration curve of silica–CMD material consisted of three linear areas. The first area of linearity decrease in conductivity corresponded to the neutralisation of H^+^ ions from excess HCl. The second area increased in conductivity and represented the neutralisation the CMD carboxyl groups, whilst the third area of linearity demarcated the point of complete neutralisation of the CMD material by a continued increase in conductivity due to addition of excess NaOH. The ΔV for NaOH in the second part of the titration was used to calculate the number of moles of available -COOH and was determined via computing the first derivatives of the titration curve seen in Fig. 4b, c where ΔV for NaOH was calculated from *V*_2_ to *V*_1_. The titration of seven individual batches coated CMD silica resulted in an average of 0.60 ± 0.01 mmol/g, moles of -COOH groups per gramme of material (2% RSD). For comparison, a control conductometric titration of aminopropyl silica was performed which produced an expected “v”-shaped conductivity graph (see Fig. S3).

### μSPEed trypsin reactor

The compatibility and covalent immobilisation of biomolecules on the CMD material were evaluated using horse radish peroxidase (HRP) as a model enzyme. HRP was immobilised in situ (within the μSPEed cartridges) to the CMD/silica support material harnessing EDC/NHS crosslinking chemistry [29, 30]. HRP catalyses the reaction of azino-bis(3-ethylbenzothiazoline-6-sulfonic acid) (ABTS) in the presence of H_2_O_2_ to a radical cation with absorbance at 620 nm. The formation of the product was therefore monitored via spectroscopy to investigate the stability and conversion rate of the IMER. Figure S2 shows the absorbance of the ABTS radical cation, confirming the activity of HRP after immobilisation. The same IMER was employed for ten individual reaction cycles demonstrating that the conversion of ABTS was stable with an average value of 0.226 ± 0.018 AU and a RSD of 8%. Similarly, a non-covalent immobilisation was performed using the above colorimetric assay resulting in a decreasing signal across the ten measurements, indicating leaching of HRP from the support material (see Fig. S2). Between each of the ten readings, a mild wash buffer of 10 mM MESS was used. However, after the tenth reading, the cartridges were washed with a PBS (1×) pH 7.4 buffer solution, resulting in no absorbance signal at 620nm for the non-covalently bound HRP indicating complete removal of HRP from the cartridge. The same washing buffer was used for the covalently bound HRP without detrimental effects.

**Fig. 5:**
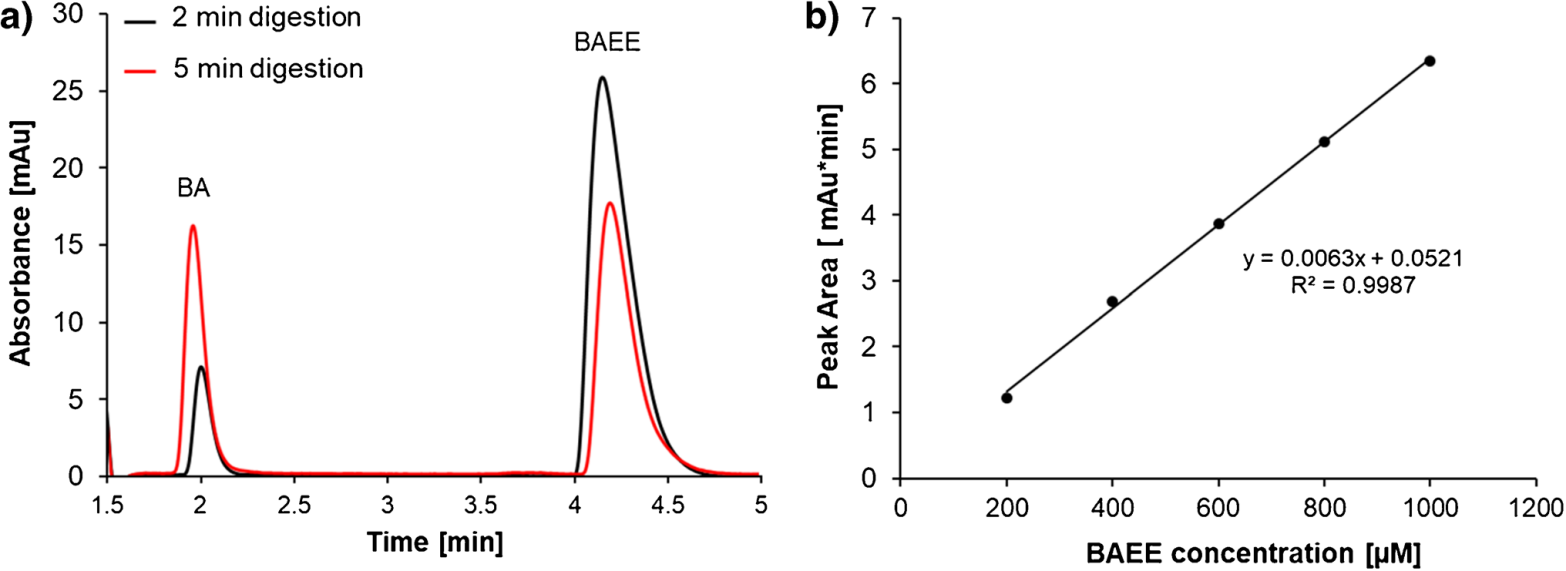
**a** HPLC-UV chromatograms at 254 nm for BAEE digestion to BA for a 2-min digest (black line) and 5-min digest (red line). **b** Calibration curve constructed for BAEE analysis after 2-min digestion by HPLC-UV

The same crosslinking chemistry was used to immobilise trypsin onto the generic CMD μSPEed cartridge. The immobilised trypsin activity and repeatability within the cartridge was evaluated using BAEE as a model compound [31, 32]. In the presence of trypsin, BAEE produced N_α_-benzoyl-l-arginine (BA) and ethanol via cleavage at the carboxyl side of arginine as seen in Fig. 5. This cleavage was monitored using high-performance liquid chromatography (HPLC) with a UV detector at 254 nm*.* The incubation time was evaluated at 2-min and 5-min digestions of 1000 μM BAEE. Chromatographic signals for the undigested BAEE and the digest product (BA) were detected at 4 and 2 min, respectively. The peak ratio area of BA/BAEE was calculated for each digest where the 2-min digest resulted in a ratio of 0.10 compared to 0.33 for the 5-min digest, showing that increased incubation time resulted in more product formation. Similarly*,* the influence of flow rate was also assessed. Whilst keeping the BAEE substrate incubation time the same between two cartridges, two flow rates of 300 and 600 μL/min were evaluated. The obtained BA/BAEE peak area ratio was 0.10 and 0.11 for 300 μL/min and 600 μL/min, respectively, demonstrating that the digestion efficiency was not affected by flow rates but incubation time.

The trypsin cartridge digestion reproducibility was evaluated by preparing a calibration curve (see Fig. 5b) to calculate the concentration of BAEE remaining after a 2-min incubation digest of 1000 μM BAEE within six trypsin cartridges. The concentration of BAEE for the six samples averaged 513 ± 26 mol/L with a RSD of 5.2% indicating reproducible digestion.

**Fig. 6:**
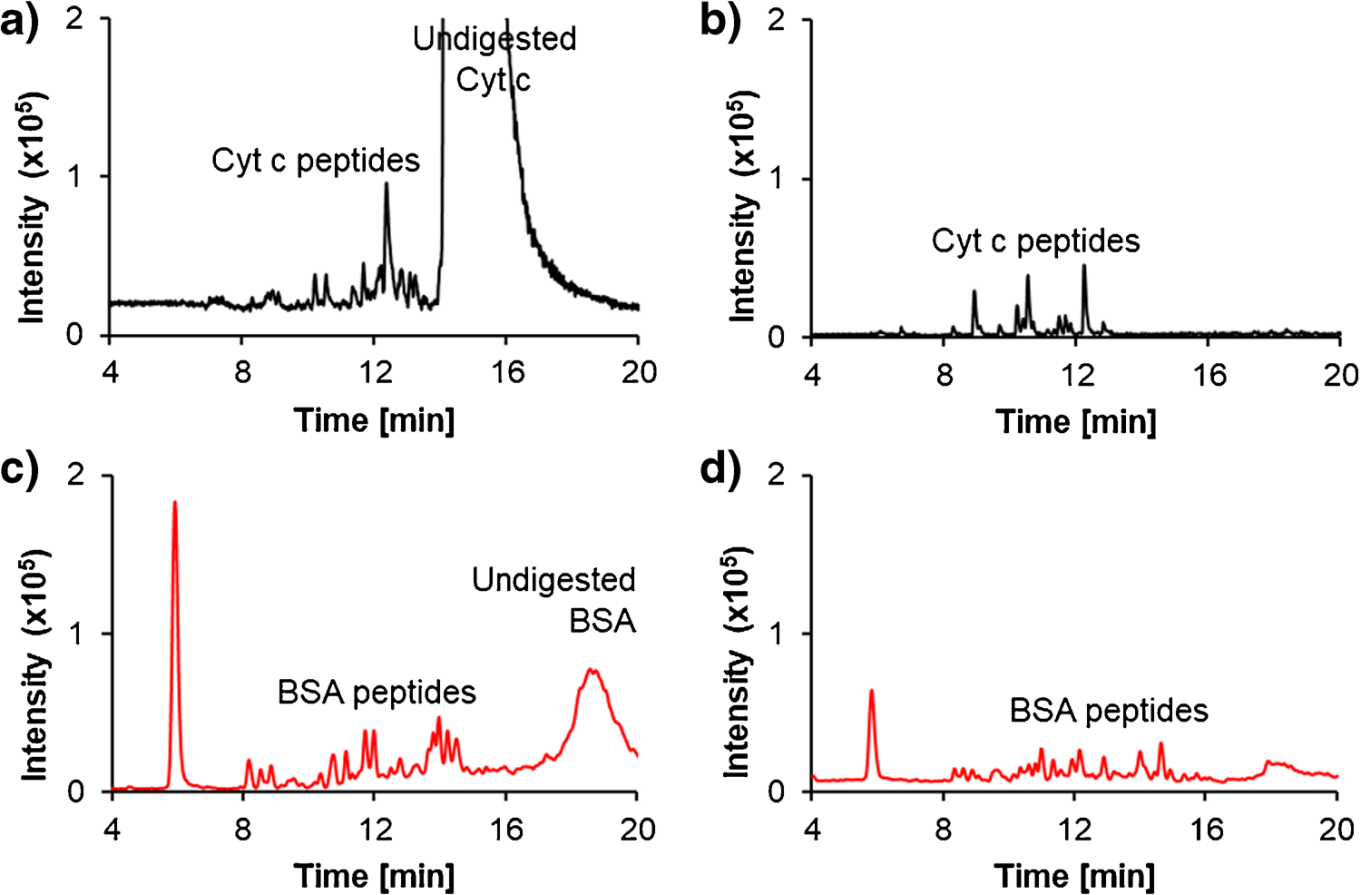
Total ion chromatograms (TIC) acquired by LC-QToF-MS after tryptic digestions of Cyt c and BSA. Different protein loads and incubation times were tested to determine the optimal working range of the developed IMER. **a** A 5-min digest of 10 μg of Cyt c, **b** a 5-min digest of 1 μg Cyt c, **c** a 10-min digest of 5 μg BSA and **d** a 10-min digest of 1 μg BSA

Following trypsin activity experiments, preliminary protein loading capacity and digestion incubation times were investigated by loading Cyt c and BSA from 1 to 10 μg for 5 and 10 min, respectively. Figure 6 shows the total ion chromatograms (TICs) from LC-QToF-MS analysis after tryptic digestion within the IMER. Initially, a 5-min room temperature digestion of Cyt c was attempted with 10 μg of Cyt c loaded onto the cartridge. There was a large peak at 16 min representing undigested Cyt c (Fig. 6a). Loading of 1 μg of Cyt c under the same conditions resulted in complete digestion of the protein with no evidence of residual intact Cyt c (Fig. 6b). Similarly, loading of 10 μg of BSA and after 10 min of incubation, a large peak of undigested BSA was observed at 18 min (Fig. 6c). Reducing the protein load to 1 μg resulted in improved digestion and adequate peptide coverage (Fig. 6d). Therefore, efficient digestion of these two proteins required ≤ 1 μg, and higher amounts should be avoided to prevent IMER overloading.

### μSPEed IMER reproducibility and application to model proteins

Following optimisation of the μSPEed workflow for protein digestion, three trypsin cartridges were prepared to assess the repeatability of the μSPEed reactor for BSA digestion. As before, each cartridge was loaded with 1 μg of reduced and alkylated BSA with a 10-min incubation time (30 cycles of 100 μL sample at 300 μL/min) and analysed using LC-LTQ-Orbitrap MS. The average sequence coverage of BSA was 79 ± 4% demonstrating that the IMERs were suitable for incorporation into proteomic workflows.

Additionally, the performance of the IMER to digest and identify proteins by their peptide fragmentation fingerprint was further evaluated by digestion of Cyt c. The IMER digestion was compared to the common 18-h in-solution digestion method at 37 °C and both samples analysed by LC-LTQ-Orbitrap MS. Prior to analysis, all samples were diluted appropriately to ensure the same starting protein amount for digestion was performed across both methods. The IMER performance for the digestion of Cyt c was comparable with in-solution digestion obtaining protein sequence coverages of 80% and 87%, respectively.

### Preparation of the immunosorbent for protein isolation

Anti-BSA and BSA protein were used to develop the immunoaffinity extraction μSPEed cartridge. To ensure covalent immobilisation of the antibody onto silica–CMD material and Ab–Ag formation, a colorimetric visualisation using HRP and ABTS was performed following the scheme described in Fig. 1a, b, respectively. A turquoise colour was observed for the covalently bound anti-BSA indicating successful immobilisation to the silica–CMD material. Similarly, the Ab–Ag formation was assessed by covalently immobilised BSA onto the support material. This resulted in a turquoise colour solution demonstrating the successful formation of the anti-BSA and BSA complex (see Table S1). For both experiments, control analyses were performed where no colour change was observed.

### Immunoaffinity extraction followed by enzymatic digestion

The μSPEed immunoaffinity cartridge and trypsin micro-reactor were combined for an automated method for protein isolation and digestion. Using the ePrep Workstation, samples, reagents and buffers for both cartridges were placed on the workstation bed and the immunoaffinity extraction and digestion methods setup to run sequentially, thus creating an automated workflow method combining the two cartridges. The respective cartridges were prepared according to Fig. S4 where following immunoaffinity isolation, the eluted fraction was diluted and buffered to pH 8 for compatibility with trypsin digestion. BSA in a concentration of 20 ng/μL was used as model protein, and once isolated and digested, the obtained peptide fragments were analysed by LC-LTQ-Orbitrap MS. The average sequence coverage for BSA was 87 ± 1% (*n*=3) demonstrating the efficiency of the developed workflow for protein isolation followed by tryptic digestion (see Table 1, 1st use of affinity cartridge).

**Table 1:** BSA protein coverage after immunoaffinity extraction followed by tryptic digestion using the affinity cartridge one and two times

IMER	Protein coverage (1st use of affinity cartridge)	Protein coverage (2nd use of same affinity cartridge)
1	88%	86%
2	86%	96%
3	86%	81%

After immunoextraction, the cartridge was immediately reconditioned to pH 8 twice with 500 μL of wash buffer to prevent antibody denaturation for further use. The regeneration of the antibodies allows reuse of the immunosorbent reducing the cost of the extraction procedure. The regeneration of the immunosorbent was assessed by using the same affinity cartridge twice where a BSA protein coverage of 86 ± 8% (*n*=3) was observed (see Table 1, 2nd use of same affinity cartridge). One or two uses of the same affinity cartridge led to similar protein coverages, demonstrating that the immunosorbent may be reused to limit waste and expenses.

To further assess the performance of the designed automated workflow, 20 ng/μL of BSA were spiked into a human serum sample. This sample was reduced and alkylated prior to analysis. The affinity μSPEed cartridge was used for the isolation of the spiked BSA followed by digestion and analysis by LC-LTQ-Orbitrap MS. BSA average sequence coverage was 25 ± 1% (*n*=3) (see Fig. S5). The sequence coverage obtained was sufficient for the identification of the BSA present in the serum sample, clearly demonstrating the potential of the workflow to isolate the protein of interest from a complex matrix followed by a successful tryptic digestion of the isolated protein. 

The developed IMERs present easy handling, automation and high reproducibility. Moreover, in comparison with the commonly used pre-immobilisation strategy, our modified silica–CMD material is packed into the μSPE cartridges allowing in-situ immobilisation. This offers new avenues for the immobilisation of other enzymes (e.g. pepsin or chymotrypsin) and antibodies which will become useful for various micro-reactor strategies.

## Conclusions

In this study, we developed a new customised support material based on silica particles coated with carboxymethylated dextran (CMD) using an EDC/NHS crosslinking chemistry. This new material was characterised using FTIR, SEM, conductometric titrations and enzymatic colorimetric assays. The developed trypsin IMER method showed high repeatability and low relative standard deviation (RSD) of 4% protein coverage. Moreover, the IMER was suitable for rapid protein digestion within 10 min at room temperature, with similar digestion performance of Cyt c to that of an in-solution digestion performed for 18 h at 37 °C. The same functionalised support material was employed to develop an accurate and reproducible automated method for the isolation, pre-concentration and tryptic digestion of trace level proteins using customised μSPEed cartridges. BSA was used as model protein, and once isolated and digested, the solution containing the peptide fragments was analysed by LC-LTQ-Orbitrap MS. The average sequence coverage obtained for BSA was 87 ± 1% demonstrating the efficiency of the developed workflow for protein isolation followed by tryptic digestion. We also demonstrated that it is possible to reuse the immunosorbent reducing the cost of the extraction procedure. Finally, we assessed the performance of the designed automated workflow for the analysis of BSA spiked in a human serum sample, being able to successfully identify the target protein within this complex matrix.

The developed IMERs are compatible with existing sample preparation workflows and technologies and will improve sample throughput and comparability. Furthermore, the automated in-situ immobilisation offers further opportunities for immobilisation of other enzymes and biomolecules and has potential as platform for targeted protein quantification in clinical settings.

## Supplementary information

ESM 1 (DOCX 775 kb)

## Data Availability

All data generated and/or analysed during this study are included in this published article (and its supplementary information files).
