# Supplementary material for: Immunoaffinity extraction followed by enzymatic digestion for the isolation and identification of proteins employing automated μSPE reactors and mass spectrometry
Source: Anal Bioanal Chem. 2022 Nov 12;415(18):4173–84. doi: 10.1007/s00216-022-04381-0 (PMC10328895; doi:10.1007/s00216-022-04381-0)
Supplement: Supplementary file 1 — (DOCX 775 kb) [file 216_2022_4381_MOESM1_ESM.docx]

Supplementary material

**Immunoaffinity extraction followed by enzymatic digestion for the isolation and identification of proteins employing automated μSPE reactors and mass spectrometry**

Karen Duong^1^, Simin Maleknia^2^, David Clases^1,3^, Andrew Minett^4^, Matthew P. Padula^5^, Philip A. Doble^1^ and Raquel Gonzalez de Vega^1,6^*

^1^ *The Atomic Medicine Initiative, University of Technology Sydney*

^2^ *School of mathematics and Physical Sciences, Faculty of Science, University of Technology Sydney*

*^3^ Nano Micro LAB, Institute of Chemistry, University of Graz, Austria*

^4^ *ePrep Pty Ltd, Oakleigh, Victoria, 3166, Australia*

^5^ *School of Life Sciences and Proteomics Core Facility, Faculty of Science, University of Technology Sydney*

*^6^TESLA-Analytical Chemistry, Institute of Chemistry, University of Graz, Austria*

*Corresponding author: [raquel.gonzalez-de-vega@uni-graz.at](mailto:raquel.gonzalez-de-vega@uni-graz.at)

**Table of contents**

- **Figure S1** Instrumental set up for sample preparation
- **Figure S2** Covalent and non-covalent immobilisation of HRP onto modified support material
- **Figure S3** Control conductometric titration of aminopropyl silica
- **Figure S4** Automated μSPEed workflows for protein isolation followed by tryptic digestion
- **Figure S5** BSA protein coverage by LC-LTQ-Orbitrap MS of the spiked human sample
- **Table S1** Covalent antibody immobilisation onto support material and Ab-Ag complex formation

**Figure S1** a) digiVOL^®^ syringe driver used for method development and the press-fit µSPEed cartridge onto a syringe. The µSPEed offers two flow paths where solutions are aspirated through the one-way valve and dispensed onto the sorbent packing material; b) ePrep Sample Preparation Workstation.


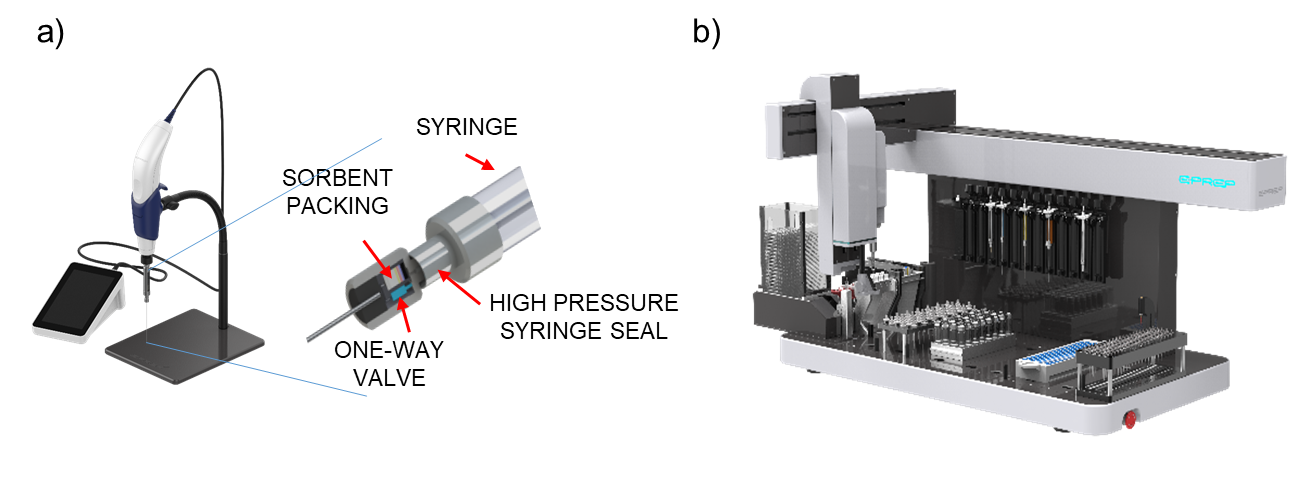


**Figure S2** Absorbance of ABTS radical cation at 620 nm for covalent (red) and non-covalent (blue) immobilisation of HRP onto Silica-CMD material


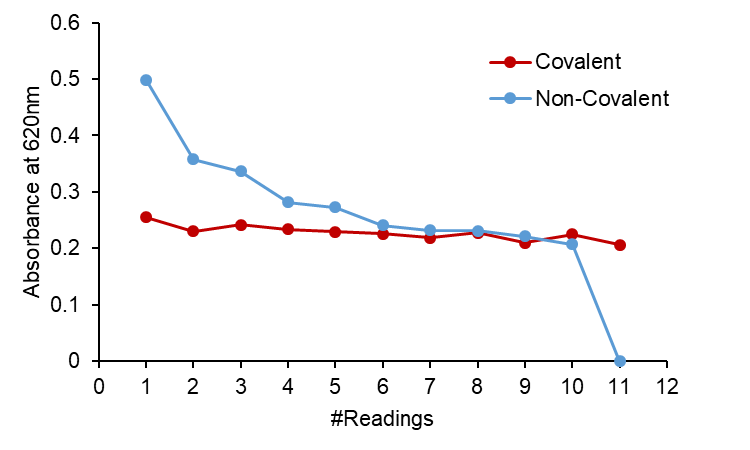


**Figure S3** Control conductometric titration of aminopropyl silica


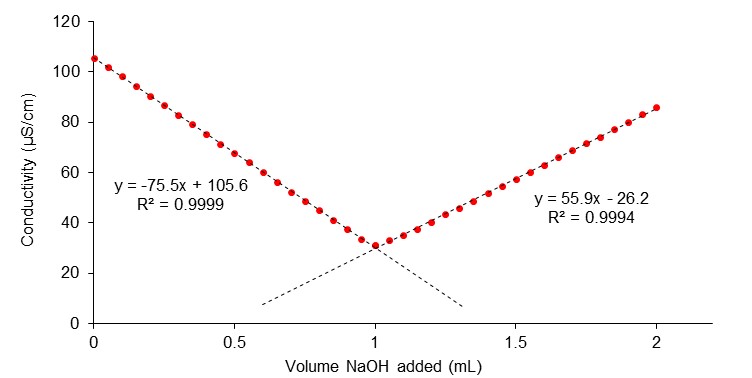


**Figure S4** Each step for the operation of the trypsin IMER was empirically optimised by evaluation of various elution, washing and running buffers, incubation times and flow rates. The immobilisation and digestion process were developed to ensure rapid and efficient proteolytic digests. A salt buffer wash was optimised to ensure removal of non-specific binding of the immobilised ligand. At relatively high salt concentrations (10 mM CaCl_2_) non-specifically bound trypsin enzyme was washed from the cartridge. The running buffer was also optimised for maximum ligand activity. Tris-HCl with CaCl_2_ salt is commonly used in digestion protocols as it has been reported in literature that Ca_2_+ ions stabilise the trypsin structure resulting in preserved enzyme activity [1] and prevent denaturation of the enzyme. The elution buffer was optimised to ensure all digested peptides were eluted from the cartridge prior to LC-MS analysis and consisted of 25 mM Tris, 10 mM CaCl_2_ and 10% ACN.

Similarly, as immunoaffinity extraction aimed to reduce sample matrix complexity, it was important that unwanted non-specific components are washed from the cartridge after sample loading. Non‑specifically bound components may interact with the surface support through several interactions such as Van der Waals forces and electrostatic forces depending on the physiochemical properties. Therefore, a wash buffer must be of a suitable composition to disrupt these forces and remove them from the cartridge. Non‑specifically bound protein can be removed using high salt concentration buffers such as PBS (1x) solution. This was because the silica-CMD material exhibited electrostatic forces that interacted with charged amino acid residues found in proteins and antibodies. However, salt solutions containing a high ionic strength can also disrupt the Ab‑Ag protein‑protein binding interactions [2]. Therefore, the salt wash buffer was optimised to have sufficient ionic strength to remove non‑specific binding components whilst mild enough to not disrupt the Ab‑Ag interaction and consisted of 25 mM Tris, 20 mM NaCl. Optimised and automated μSPEed workflow for protein isolation (A) and digestion (B) are shown in this figure.

**Figure S5** BSA protein coverage (26%) obtained by LC-LTQ-Orbitrap MS of the spiked human sample after protein isolation followed by tryptic digestion.


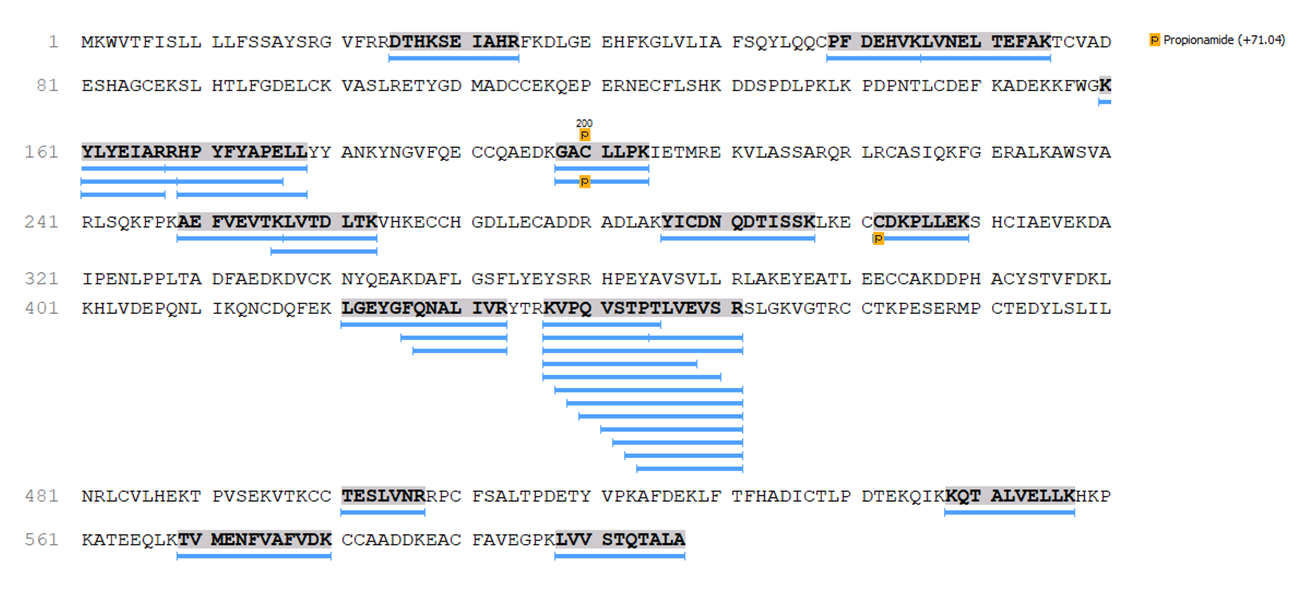


**Table S1** Covalent antibody immobilisation onto support material and Ab-Ag complex formation following diagram on Figure 1.

| Anti-BSA immobilisation | Control |
| --- | --- |
| 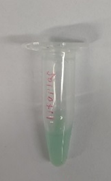 | 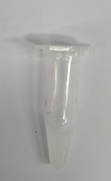 |
| BSA immobilisation & Ag-Ab complex formation | Control |
|  | **** |

1. Kotormán M, Laczkó I, Szabó A, Simon LM (2003) Effects of Ca2+ on catalytic activity and conformation of trypsin and α-chymotrypsin in aqueous ethanol. Biochem Biophys Res Commun 304:18–21 . doi: 10.1016/S0006-291X(03)00534-5

2. Ayyar BV, Arora S, Murphy C, O’Kennedy R (2012) Affinity chromatography as a tool for antibody purification. Methods 56:116–129 . doi: 10.1016/j.ymeth.2011.10.007
